# RbAp48 expression and neuronal damage in the gerbil hippocampus following 5 min of transient ischemia

**DOI:** 10.1186/s42826-019-0011-3

**Published:** 2019-07-31

**Authors:** Joon Ha Park, Tae-Kyeong Lee, Dae Won Kim, Cheol Woo Park, Young Eun Park, Bora Kim, Jae-Chul Lee, Hyang-Ah Lee, Moo-Ho Won, Ji Hyeon Ahn

**Affiliations:** 1 Department of Biomedical Science, Research Institute of Bioscience and Biotechnology, Hallym University, Chuncheon, Gangwon 24252 Republic of Korea; 2 Department of Neurobiology, School of Medicine, Kangwon National University, Chuncheon, Gangwon 24341 Republic of Korea; 3 Department of Biochemistry and Molecular Biology, and Research Institute of Oral Sciences, College of Dentistry, Gangnung-Wonju National University, Gangneung, Gangwon 25457 Republic of Korea; 4 Department of Obstetrics and Gynecology, School of Medicine, Kangwon National University, Chuncheon, Gangwon 24341 Republic of Korea

**Keywords:** Astrocytes activation, delayed neuronal death, RbAp48, Transient ischemia

## Abstract

Histone-binding protein RbAp48 has been known to be involved in histone acetylation, and epigenetic alterations of histone modifications are closely associated with the pathogenesis of ischemic reperfusion injury. In the current study, we investigated chronological change of RbAp48 expression in the hippocampus following 5 min of transient ischemia in gerbils. RbAp48 expression was examined 1, 2, 5, and 10 days after transient ischemia using immunohistochemistry. In sham operated gerbils, RbAp48 immunoreactivity was strong in pyramidal and non-pyramidal cells in the hippocampus. After transient ischemia, RbAp48 immunoreactivity was changed in the cornu ammonis 1 subfield (CA1), not in CA2/3. RbAp48 immunoreactivity in CA1 pyramidal neurons was gradually decreased and not detected at 5 and 10 days after ischemia. RbAp48 immunoreactivity in non-pyramidal cells was maintained until 2 days post-ischemia and significantly increased from 5 days post-ischemia. Double immunohistofluorescence staining revealed that RbAp48 immunoreactive non-pyramidal cells were astrocytes. At 5 days post-ischemia, death of pyramidal neurons occurred only in the CA1. These results showed that RbAp48 immunoreactivity was distinctively altered in pyramidal neurons and astrocytes in the hippocampal CA1 following 5 mins of transient ischemia. Ischemia-induced change in RbAp48 expression may be closely associated with neuronal death and astrocyte activation following 5 min of transient ischemia.

## Introduction

Ischemic stroke can cause ischemic reperfusion injury including comprehensive process with vascular leakage, cell death process, transcriptional reprogramming and immune activation [1]. Recent studies have shown that posttranslational (epigenetic) alterations including histone modifications or DNA methylation in injured brain tissues by ischemic insults are closely involved in the pathogenesis of reperfusion injury [2].

Histone acetylation destabilizes histone-DNA interaction and recruits activators or inhibitors of gene transcription [3]. In general, increased histone acetylation is associated with gene transcriptional activation, whereas deacetylation is accompanied by repression of gene transcription [4]. The acetylation and deacetylation of histone is generally regulated by histone acetyltransferases (HATs) and histone deacetylases (HDACs) [5]. Recent studies have suggested that inhibition of histone deacetylase shows neuroprotective effects in brain ischemia [6, 7].

Histone-binding protein RbAp48 is a member of chromatin-remodeling complexes, such as nucleosome remodeling and deacetylase (NURD) complex proteins which contain histone deacetylases, chromodomain helicase DNA-binding proteins, methyl-CpG-binding domain and metastasis associated gene [8–10]. RbAp48 is required for transcriptional repression mediated by HDACs [11]. In addition, RbAp48 is involved in the regulation of pluripotency, differentiation and cell cycle genes in multiple human pluripotent stem cells [12]. This RbAp48 function is closely related to memory consolidation, and the deficiency of RbAp48 in the hippocampus is a major factor of age-related memory loss [13, 14].

Although many previous studies have reported age-related change in RbAp48 expression in the hippocampus and its associated memory function, very little is known about changes in RbAp48 expression and its role in the hippocampus following transient ischemia. Therefore, in this study, we investigated time-dependent change and expression pattern of RbAp48 the hippocampus following 5 min of transient ischemia in gerbils.

## Material and method

### Experimental animals

Male Mongolian gerbils (*Meriones unguiculatus*), aged 6 months (body weight, approximately 68–73 g), were obtained from the Experimental Animal Center, Kangwon National University, Chuncheon, Republic of Korea. They were maintained at a constant temperature (23 ± 0.3 °C) and humidity (50 ± 0.5%) with a 12-h light/dark cycle. The process of handling and caring animals conformed to the guidelines of the current international laws and policies (NIH Guide for the Care and Use of Laboratory Animals, The National Academies Press, 8th Ed., 2011). Experimental procedure for this study was approved by the Institutional Animal Care and Use Committee at Kangwon National University (approval number: KW-180124-1). In this study, we minimized numbers of gerbils used and the suffering caused by the procedure used in this experiment.

### Induction of transient ischemia

As described in our previous studies (Lee and others 2010b; Park and others 2014; Park and others 2016), the induction of transient ischemia was performed as follows. In brief, gerbils were anesthetized with a mixture of isoflurane (2.5%) in oxygen (33%) and nitrous oxide (67%). A midline incision was made on the ventral surface of the neck, and bilateral common carotid arteries were exposed and occluded for 5 min by using aneurysm clips. Body temperature (37 ± 0.5 °C) was controlled with a rectal temperature probe (TR-100; Fine Science Tools, Foster City, CA, USA) by using a thermometric blanket during and after transient ischemia. Sham operated gerbils received the same procedure without bilateral common carotid artery occlusion.

### Preparation of histological sections

For immunohistochemical staining, cresyl violet (CV) staining, Fluoro-Jade B (FJB) histofluorescence and double immunofluorescence staining, brain sections containing the hippocampus were prepared from the sham and ischemia operated gerbils (*n* = 7 at each point in time) at designated times (6 h, 1 day, 2 days, 5 days and 10 days after transient ischemia). As described in our published papers [15–17], histological sections were prepared as follows. In short, the gerbils were anaesthetized with pentobarbital sodium (60 mg/kg, JW Pharmaceutical Co., Ltd., Seoul, Korea) and perfused transcardially with 0.1 M phosphate-buffered saline followed by 4% paraformaldehyde. And then, their brains were removed and post-fixed with the same fixative for 8 h. The fixed brains were cryoprotected by infiltration with 30% sucrose for 9 h and serially sectioned into 30-μm coronal sections in a cryostat (Leica, Wetzlar, Germany).

### Immunohistochemistry

Immunohistochemical staining was carried out for RbAp48 according to our published protocol [15–17]. Briefly, the sections were incubated with rabbit anti-RbAp48 (1:250, abcam, Cambridge, MA, USA) as primary antibody. The RbAp48-reacted sections were exposed to biotinylated goat anti-rabbit IgG (1:250, Vector, Burlingame, CA, USA) and streptavidin peroxidase complex. Finally, the reacted sections were visualized with 3,3′-diaminobenzidine. In order to confirm the specificity of RbAp48 immunoreaction, negative control test was done by using pre-immune serum instead of RbAp48 antibody. The negative control test showed no RbAp48 immunoreactivity in immunostained tissues.

For quantitative analysis of RbAp48 immunoreactivity, 8 sections were selected with 120-μm interval in each gerbil. RbAp48 immunoreactive image in the hippocampus was captured with an AxioM1 light microscope (Carl Zeiss, Germany) equipped with a camera (Axiocam, Carl Zeiss, Germany) connected to a PC monitor. As described in our published papers [15–17], RbAp48 image was captured in corresponding area (250 × 250 μm) of each hippocampal subfield at 20× primary magnification, and the image was calibrated into an array of 512 × 512 pixels. The density of RbAp48 immunoreactive structures was evaluated on the basis of optical density (OD). The OD was obtained after the transformation of the mean gray level by using a formula: OD = log (256/mean gray level). A ratio of OD was calibrated as % (relative optical density, ROD) by using NIH Image 1.59 software. A ratio of ROD was calibrated as %, with the sham group (100%).

### CV staining

CV (a marker for Nissl body) histochemical staining was performed to investigate cellular distribution and morphology in paraformaldehyde or formalin-fixed tissue. In brief, according to our published method [18], CV acetate (Sigma, MO, USA) was dissolved (1%) in distilled water, and glacial acetic acid was added to this solution. The sections in each group were stained with CV solution, differentiated in 95% ethyl alcohol for 2–30 min and check microscopically for best result. Finally, the differentiated sections were dehydrated with serial ethanol and coverslipped.

CV stained sections in the hippocampus was observed under an AxioM1 light microscope (Carl Zeiss, Germany) equipped with a camera (Axiocam, Carl Zeiss, Germany) connected to a PC monitor.

### Fluoro jade B (FJB) histofluorescence staining

FJB (a marker for neurodegeneration) histofluorescence staining was performed to examine ischemia-induced neuronal death. As previously described [15–17], in brief, the sections in each group were serially stained with a 1% sodium hydroxide solution, a 0.06% potassium permanganate solution and a 0.0004% FJB (Histochem, Jefferson, AR, USA) solution. The sections on slides were placed on a slide warmer, set at approximately 50 degrees C, until they were fully dry for 5–10 min. Finally, the dry slides were cleared by immersion in xylene and coverslipped.

Numbers of FJB positive cells as dead cells were analyzed according to our published method [17]. In brief, we selected 8 sections from each animal with 120-μm interval according to AP (Antero-posterior) − 1.4 to − 2.2 mm of the gerbil brain atlas. Images of FJB positive cells were captured by using an epifluorescent microscope (Carl Zeiss, Göttingen, Germany) equipped with a blue excitation light (450–490 nm) and a barrier filter. Cells were obtained in a 250 × 250 μm square, and cell count was obtained by averaging the total number of FJB positive cells from each animal per group by using an image analyzing system (software: Optimas 6.5, CyberMetrics, Scottsdale, AZ).

### Double immunofluorescence staining

To examine types of cells containing RbAp48 immunoreactivity, double immunofluorescence staining was performed according to our published protocol (Park and others 2016). In brief, we used rabbit anti-RbAp48 (1:100, abcam, Cambridge, MA, USA)/mouse anti-GFAP (1:400, abcam, Cambridge, MA, USA) for astrocytes or goat anti-Iba1 (1:400, abcam, Cambridge, MA, USA) for microglia. The sections were incubated in the mixture of the antisera, and the incubated sections were reacted in a mixture of both donkey anti-rabbit IgG, Alexa Fluor 546 or 546 (1:500, Invitrogen, Waltham, MA, USA) and goat anti-mouse IgG, Alexa Fluor 488 (1:500, Invitrogen, Waltham, MA, USA) and donkey anti-goat IgG, Alexa Fluor546 (1:500, Invitrogen, Waltham, MA, USA).

The double immunoreaction for RbAp48/astrocytes or RbAp48/microglia was observed under a confocal MS (LSM510 META NLO, Carl Zeiss, Germany) in the Korea Basic Science Institute Chuncheon Center.

### Statistical analysis

The data shown here represent the means ± SEM. Differences of the means among the groups were statistically analyzed by analysis of variance (ANOVA) with Duncan’s post hoc test using GraphPad Instat (Instat Statistics, GraphPad Software) in order to elucidate ischemia-related differences among experimental groups. Statistical significance was considered at *P* < 0.05.

## Results

### Transient ischemia-induced change in RbAp48 immunoreactivity

**Fig. 1:**
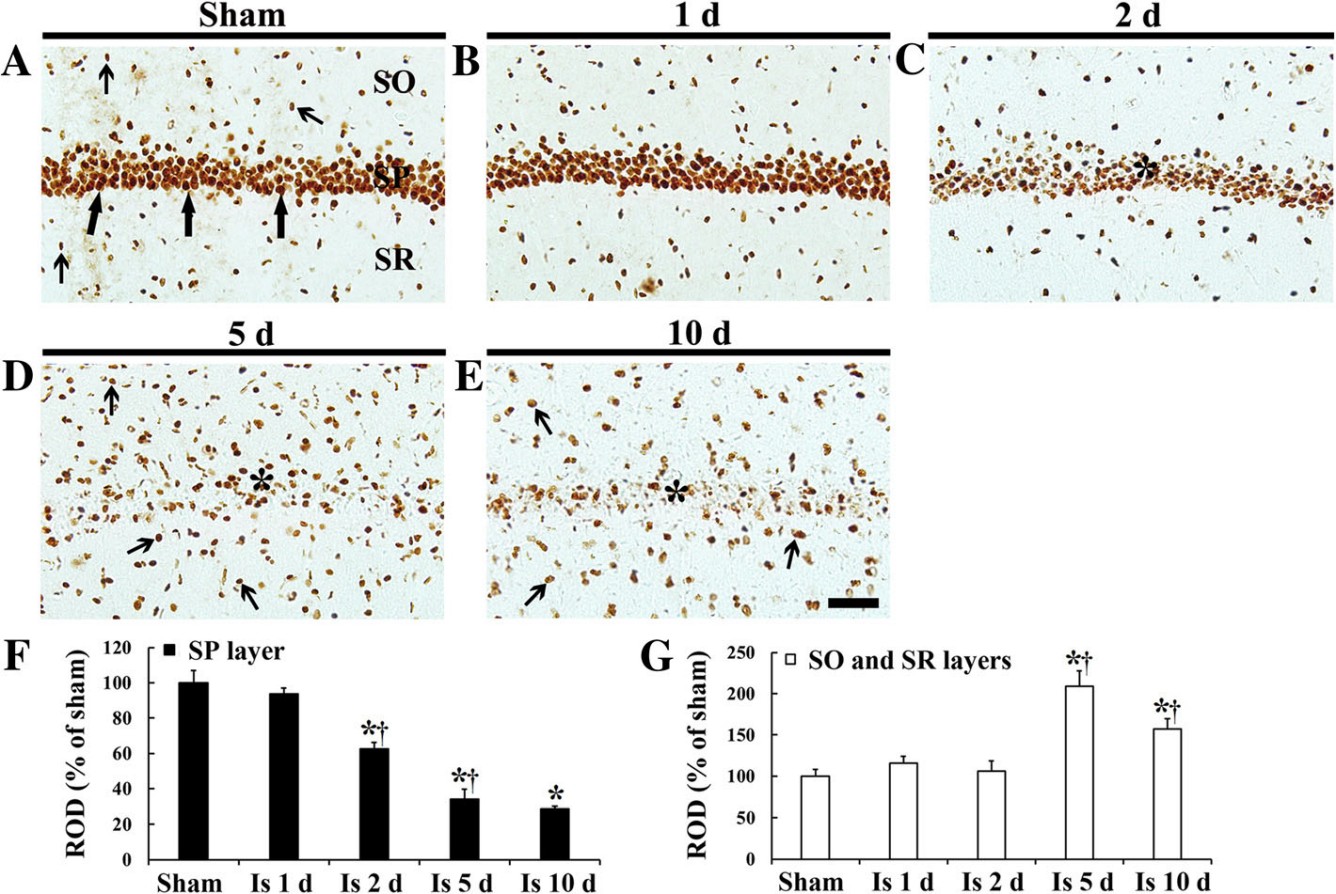
RbAp48 immunohistochemistry in CA1 of the sham operated (**a**) and ischemia operated (**b**-**e**) gerbils. In the sham operated gerbils, strong RbAp48 immunoreactivity is shown in pyramidal cells (thick arrows) and non-pyramidal cells (thin arrows). RbAp48 immunoreactivity is decreased in pyramidal cells at 2 days post-ischemia. At 5 days and 10 days post-ischemia, RbAp48 immunoreactive pyramidal cells (asterisks) were few; however, RbAp48 immunoreactive non-pyramidal cells (thin arrows) are significantly increased in numbers. SO, stratum oriens; SP, stratum pyramidale; SR, stratum radiatum. Scale bar = 50 μm. (F, G) ROD as % of RbAp48 immunoreactive structures in the SP (**f**), and in the SO and SR (**g**) after transient ischemia (*n* = 7 at each point in time, ^*^P < 0.05, significantly different from the sham operated group, ^†^*P* < 0.05, significantly different from the pre-time point group). Bars indicate the means ± SEM

In the sham operated group, strong RbAp48 immunoreactivity was predominantly observed in nuclei of pyramidal neurons in the stratum pyramidale and non-pyramidal cells in strata oriens and radiatum in the hippocampus (CA1-CA3) (Fig. 1a).

In the ischemia operated groups, RbAp48 immunoreactivity in the hippocampus was altered in CA1 (Fig. 1b-e), not in CA2 and CA3 (data not shown). At 1 day after transient ischemia, RbAp48 immunoreactivity was similar to that in the sham operated group (Fig. 1b). At 2 days after transient ischemia, RbAp48 immunoreactivity was significantly decreased in the stratum pyramidale (Fig. 1c, f). At 5 days after transient ischemia, RbAp48 immunoreactivity in the stratum pyramidale was more significantly decreased (about 34% of the sham operated group) (Fig. 1d, f); however, RbAp48 immunoreactivity was significantly increased (about 109% of the sham operated group) in non-pyramidal cells in the stratum oriens and radiatum (Fig. 1d, g). At 10 days after transient ischemia, the distribution pattern of RbAp48 immunoreactivity in ischemic CA1 was similar to that at 5 days post-ischemia (Fig. 1e, g), showing that ROD of RbAp48 immunoreactivity in non-pyramidal cells was about 85% of that at 5 days post-ischemia.

### Transient ischemia-induced neuronal death

**Fig. 2:**
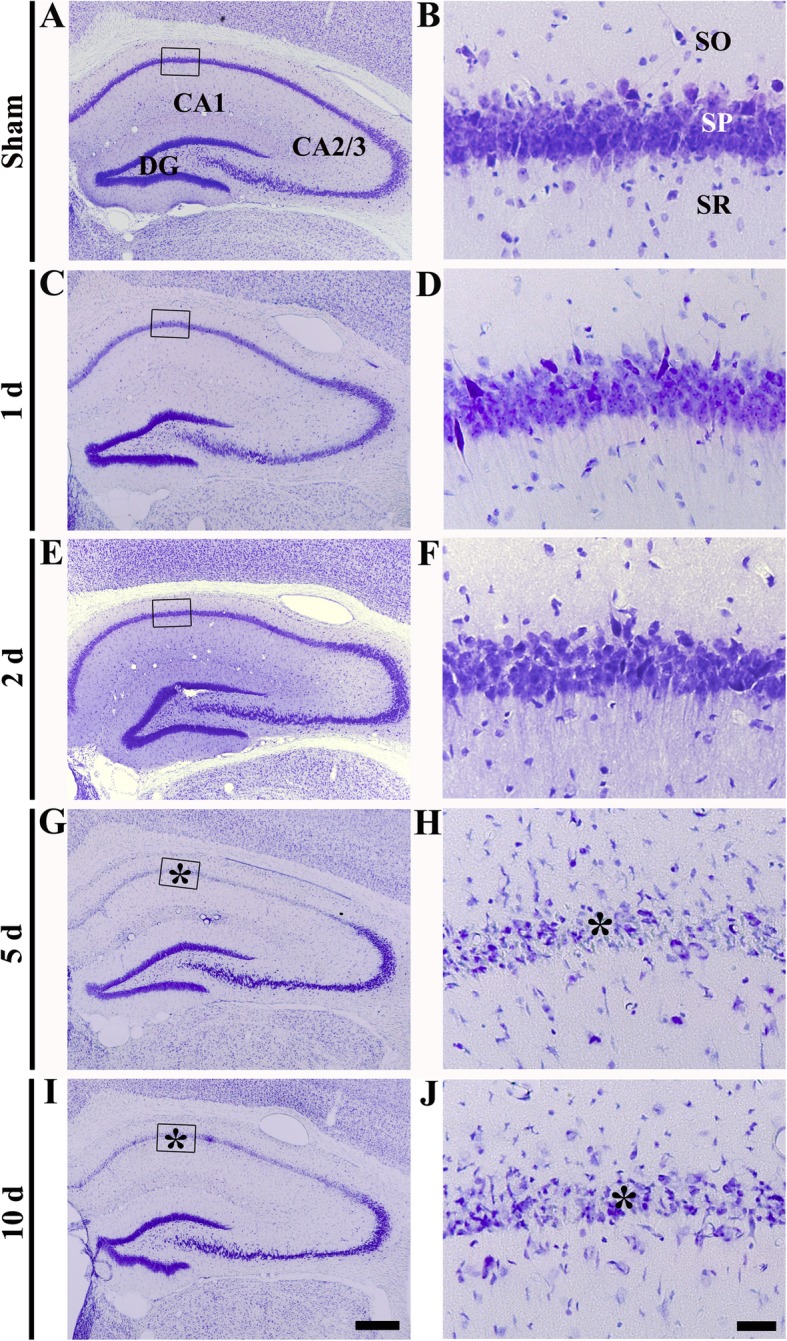
CV staining in the hippocampus of the sham operated (**a**, **b**) and ischemia operated (**c**-**j**) gerbils after transient ischemia. Low magnification of the hippocampus (**a**, **c**, **e**, **g**, **i**) and high magnification of CA1 (**b**, **d**, **f**, **h**, **j**) are presented. CV positive pyramidal cells are easily found in the stratum pyramidale (SP) in CA1-CA3 of the sham operated group. In the ischemia operated group, CV positive cells are damaged in the stratum pyramidale (asterisks) only in CA1 at 5 and 10 days after transient ischemia. DG, dentate gyrus. Scale bar = 400 (**a**, **c**, **e**, **g**, **i**) and 25 (**b**, **d**, **f**, **h**, **j**) μm

**Fig. 3:**
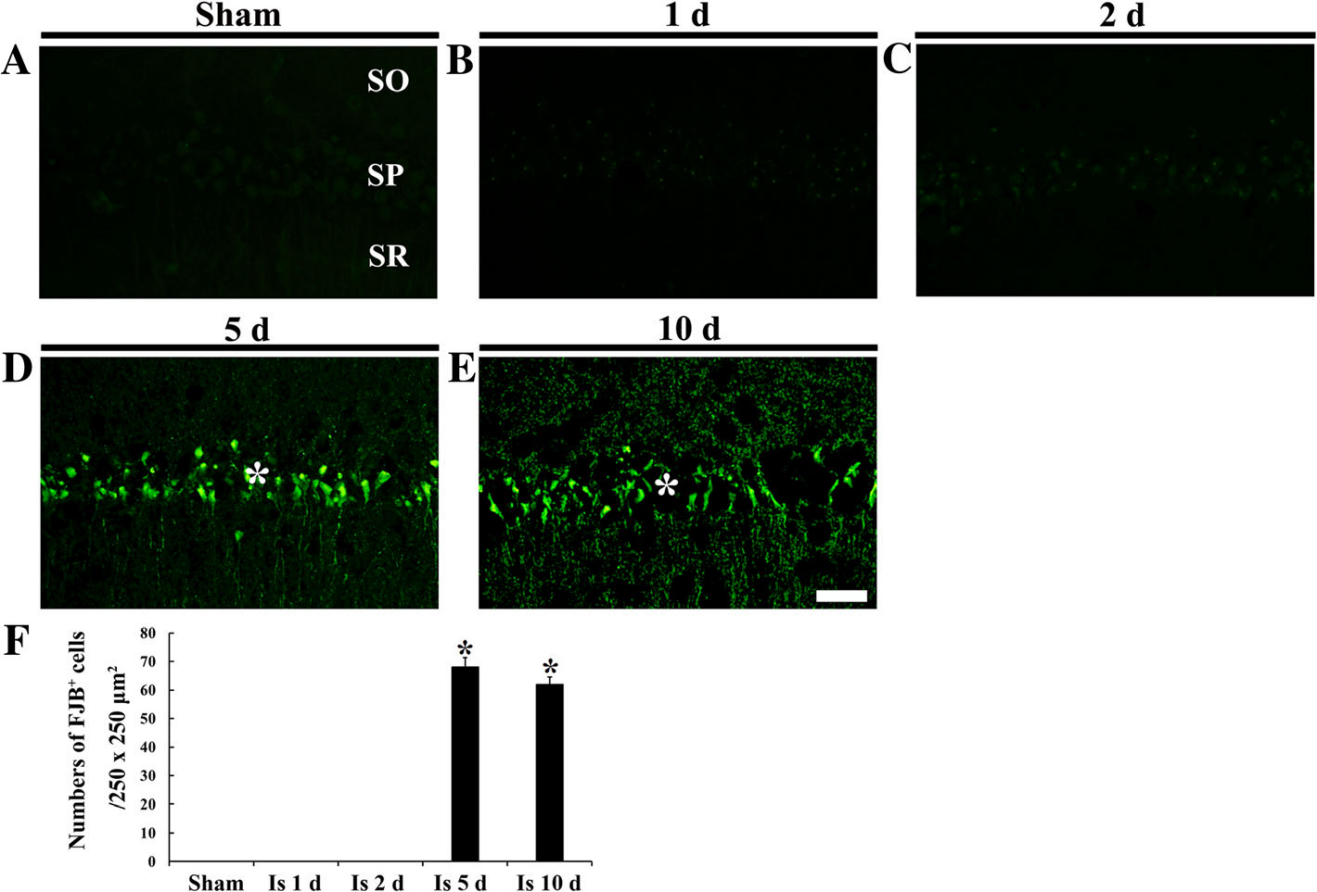
FJB histofluorescence staining in CA1 of the sham operated (**a**) and ischemia operated (**b**-**e**) gerbils after transient ischemia. FJB positive cells are not detected until 2 days after transient ischemia. However, many FJB positive cells (asterisks) are found in the stratum pyramidale (SP) at 5 and 10 days after transient ischemia. Scale bar = 50 μm. (**f**) Numbers of FJB positive CA1 pyramidal cells (*n* = 7 at each point in time, ^*^P < 0.05, significantly different from the sham operated gerbils). The bars indicate the means ± SEM

Neuronal death following 5 min of transient ischemia was examined in the hippocampus by using CV staining and FJB histofluorescence staining (Figs. 2 and 3). In the sham operated group, CV positive cells were easily found in the gerbil hippocampus (CA1-CA3), showing that large pyramidal cells consisted of the stratum pyramidale (Fig. 1a, b). In the ischemia operated groups, the distribution of CV positive cells was not altered until 2 days after transient ischemia (Fig. 2c-f). At 5 days post-ischemia, CV positive cells in the stratum pyramidale, which are named pyramidal neurons, were predominantly decreased, due to ischemic injury, in CA1, not in CA2–3 (Fig. 1g, h), showing that damage of CA1 pyramidal neurons was severer at 10 days post-ischemia (Fig. 2i, j).

FJB positive cells were not detected in any layers of CA1 in the sham operated group (Fig. 3a). In the ischemia operated groups, no FJB positive cells were shown until 2 days after transient ischemia (Fig. 3b, c). However, FJB positive cells were found in the stratum pyramidale of CA1 at 5 and 10 days after transient ischemia, and the mean number of the FJB positive CA1 pyramidal cells was significantly increased by about 68 and 62%, respectively, compared to that in the sham group (Fig. 3d-f).

### Transient ischemia-induced RbAp48 immunoreactivity in astrocytes

**Fig. 4:**
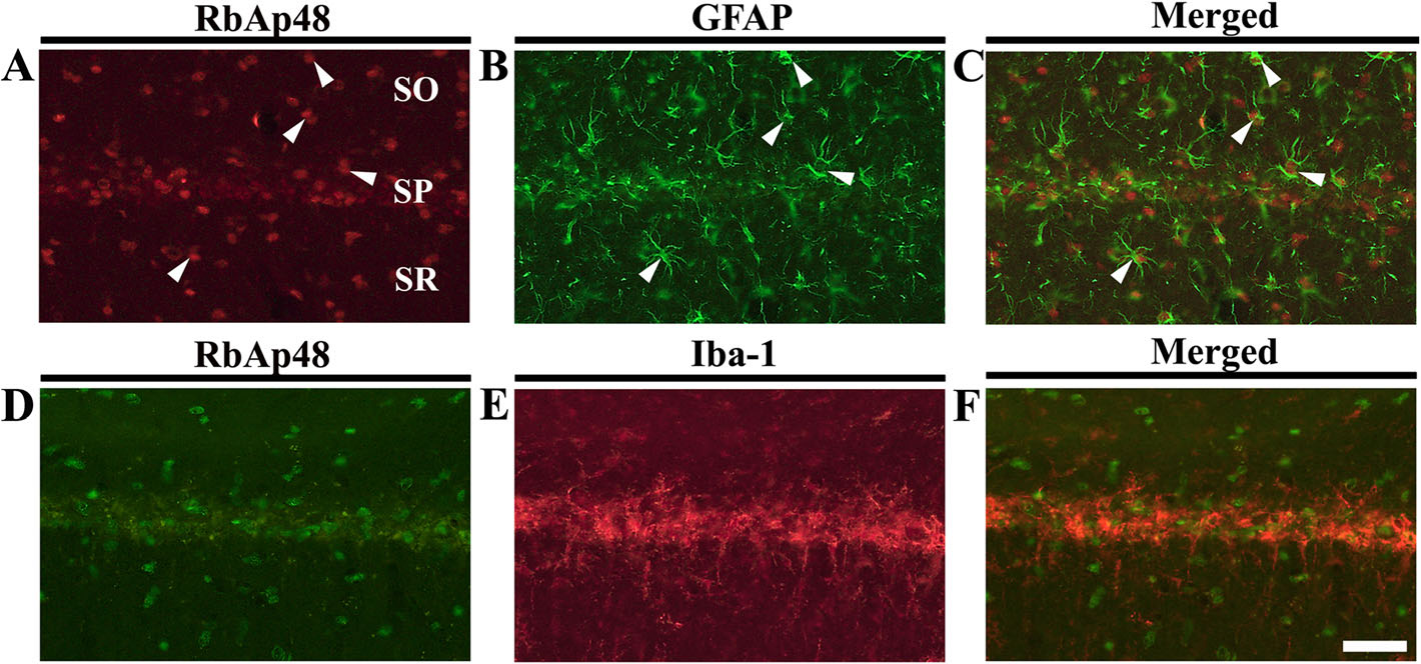
Double immunofluorescence staining for RbAp48 (red in **a** and green in **d**), GFAP (green in **b**), Iba-1 (red in **e**), and merged images (**c**, **f**) in CA1 at 10 days after transient ischemia. RbAp48 immunoreactivity is identified in GFAP-immunoreactive astrocytes (white arrowheads). Scale bar = 50 μm

RbAp48 immunoreactivity was found in non-pyramidal cells in strata oriens and radiatum in the sham operated group, and RbAp48 immunoreactive non-pyramidal cells were significantly increased at 5 and 10 days after transient ischemia (Fig. 1d, e). To identify their cell type, we conducted double immunofluorescence staining and found that RbAp48 immunoreactive cells were co-localized with GFAP positive astrocytes (Fig. 4a-c), not Iba-1 positive microglia (Fig. 4d-f) at 10 days after transient ischemia.

## Discussion

In the present study, we examined time-dependent change of histone binding protein RbAp48 immunoreactivity in the gerbil hippocampus after 5 min of transient ischemia. Our study revealed that RbAp48 immunoreactivity was expressed in both pyramidal neurons and non-pyramidal cells in the sham operated group, and RbAp48 immunoreactivity was significantly decreased in pyramidal neurons of CA1, not CA2 and CA3, from 2 days and disappeared from 5 days after transient ischemia. It has been reported that death of CA1 pyramidal neurons occurs at about 4–5 days after 5 min of transient ischemia in gerbils [19–21]. Based on these results, it is likely that ischemia-mediated change of RbAp48 expression is related to neuronal death following ischemic insults.

Hippocampal neuronal expression of RbAp48 in our study are in agreement with previous studies that showed that RbAp48 is expressed in neurons in the swine [22] and human [23] brain. As RbAp48 expression was significantly decreased or disappeared in CA1 pyramidal neurons in our study, it has been reported that a significant reduction in RbAp48 expression is revealed in the cortex of Alzheimer’s disease patients [23]. It has been demonstrated that RbAp48 exerts variety of functions. RbAp48 contributes to activation of gene transcription through histone acetylation [24] and regulates chromatin assembly in normal replication and during repair of DNA damage, so the disruption of RbAp48 induces DNA damage [25, 26]. Although we did not prove direct evidence why RbAp48 expression was reduced or disappeared in CA1 pyramidal neurons after transient ischemia in gerbils, based on previous and our current studies, it can be postulated that the decreased RbAp48 immunoreactivity in ischemic CA1 pyramidal neurons might be related to ischemia-induced changes of histone acetylation and DNA damage.

Zhang et al. [24] have reported that RbAp48 interacts with a complex of cyclic adenosine monophosphate (cAMP) response element-binding protein (CREB) binding protein (CBP), and phosphorylated CREB promotes RbAp48 binding and leads to histone acetylation during transcriptional activation (Zhang and others 2000). Yildirim et al. [27] have examined that CBP is essential in the susceptibility of ischemic neuronal death by using CBP heterozygous mutant mouse (CBP^+/−^) and demonstrated that neuroprotective effect of ischemic preconditioning against focal ischemia is mediated by increasing CBP’s acetylation activity at promoter gelsolin, a gene product which has neuroprotective properties [27]. On the other hand, CREB, a general signal-activated transcriptional mediator, is affected by ischemia. For example, some researchers have reported that global ischemia induces dephosphorylation of CREB in hippocampal CA1 neurons [28, 29], whereas increased phosphorylated CREB plays neuroprotective effect after cerebral ischemia [30, 31]. While the involvement of RbAp48 in neurons after transient ischemia has not reported, these previous studies have demonstrated that many components complexed with RbAp48 are closely involved in ischemic neuronal death. Taken together, our present findings suggest that a reduction of RbAp48 in CA1 pyramidal neurons may be related to the process of ischemic neuronal death.

In our current study, RbAp48 immunoreactivity was found in non-pyramidal cells, in the sham operated group, and RbAp48 immunoreactive in non-pyramidal cells, which were identified as astrocytes, were significantly increased in numbers from 5 days after transient ischemia. It has been reported that RbAp48 is expressed in non-neuronal cells, such as human glioblastoma cells [25] and FRO (thyroid) cells [11]. RbAp48 in FRO cells is involved in thyroid cancer proliferation (as a NF-κB effector) [11]. In addition, it has been reported that histone protein H1 is upregulated in astrocytes in the human cortex with Alzheimer’s diseases, in which chronic neuronal degeneration occurs [32]. Taken together, our current finding indicates that RbAp48 in astrocytes might be related to the proliferation of astrocytes (astrocyte activation) during ischemic reperfusion injury in the hippocampal CA1.

## Conclusions

In summary, RbAp48 immunoreactivity was apparently changed in pyramidal neurons and astrocytes in the gerbil hippocampal CA1 after 5 min of transient ischemia. These results indicate that ischemia-induced change in RbAp48 expression might be closely related to transient ischemia-induced neuronal death and astrocyte activation in transient ischemia-induced damaged regions.

## Data Availability

All data generated or analyzed during this study are included in this published article.

## Abbreviations

CA1: cornu ammonis 1 subfield
cAMP: cyclic adenosine monophosphate
CBP: CREB binding protein
CREB: cAMP response element-binding protein
CV: cresyl violet
FJB: Fluoro Jade B
HATs: histone acetyltransferases
HDACs: histone deacetylases
NURD: nucleosome remodeling and deacetylase
OD: optical density
ROD: relative optical density
